# Diet composition and selection of Père David's deer in Hubei Shishou Milu National Nature Reserve, China

**DOI:** 10.1002/ece3.9702

**Published:** 2023-01-06

**Authors:** Hao‐Lin Wang, Yue Zhao, Fei‐Jie Wang, Xin‐Jia Sun, Jian‐Qiang Zhu, Yu‐Ming Zhang, Shu‐Dong Wei, Hui Chen

**Affiliations:** ^1^ College of Life Science and Engineering Research Center of Ecology and Agricultural Use of Wetland Ministry of Education, Yangtze University Jingzhou China; ^2^ Research Center of Milu Health and Habitat Yangtze University Jingzhou China; ^3^ Administrative Office of Shishou Milu National Nature Reserve Jingzhou China; ^4^ Henan Key Laboratory of Water Pollution Control and Rehabilitation Technology Henan University of Urban Construction Pingdingshan China

**Keywords:** diet selection, *Elaphurus davidianus*, nutritional content, stable isotope

## Abstract

Hubei Shishou Milu National Nature Reserve is an ideal place to restore the wild population of Père David's deer (*Elaphurus davidianus*). Understanding foraging ecology and diet composition is essential for assessing population development or establishing long‐term effective conservation measures for endangered species. However, little is known about the diet composition of Père David's deer and its diet selection mechanism. In this study, we used stable isotope technology to investigate the diet composition of Père David's deer according to various tissues (i.e., fur, muscle, liver, heart, and feces) and seasons, and evaluated the correlation between the nutrient composition of plants and diet composition. Bayesian isotope analysis showed that the autumn and winter diet estimated by fur and fecal samples indicated a diet dominated by C_3_ grasses (42.7%–57.2%, mean), while the summer diet estimated by muscle and liver samples was dominated by C_3_ forbs (30.9%–41.6%, mean). The Pearson correlation test indicated that the contribution of winter diet composition reflected by fur and fecal samples was associated with correlations with crude protein (*r* = .666, *p* < .01) and soluble sugars (*r* = .695, *p <* .01). The results indicated that crude protein and soluble sugars were important factors influencing the winter diet selection of Père David's deer. In the context of the current reintroduction facing many challenges, such as habitat fragmentation, wetland degradation, and human disturbance, comprehensively evaluating the diet selection mechanism of Père David's deer under different resource specificities and temporal changes should be considered in the future.

## Graphical abstract

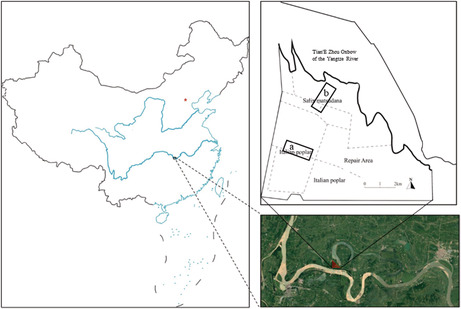

## 1 INTRODUCTION

Père David's deer (*Elaphurus davidianus*) is one of the national key protected animals belonging to Mammalia, Artiodactyla, Cervidae, and *Elaphurus* (Cao, 1985). Père David's deer became extinct in the wild in 1900 due to war, poaching, and habitat deterioration. In the 1980s, China began to implement the reintroduction project of Père David's deer and first released captive Père David's deer to nature in 1998 (Ding, 2017). With its reintroduction, ex situ conservation and population restoration have gained a great deal of attention in China (Wang et al., 2020; Xu & Yu, 2019; Xue et al., 2022).

Understanding diet composition is essential for assessing population development or establishing long‐term effective conservation measures for endangered species. However, little is known about the mechanisms of diet selection, food assimilation, and the interrelationships between Père David's deer and their habitat due to the limitations of research methods and the particularity of research objects (Ding, 2009; Hua et al., 2020; Wang & Wang, 2011). Père David's deer mainly lives in swampy and muddy areas and feeds on the young branches and leaves of grasses and some legumes (Ding, 2004). To study the diet composition of Père David's deer more systematically, Ding et al. (1989) initiated investigations of the vegetation in the territory of Père David's deer, dividing the plants into avoided, foraged, and preferred according to the frequency of feeding and the degree of foraging. Hua et al. (2020) used both fecal microanalysis and direct observation to compare and analyze the feeding habits of Père David's deer in winter in Yancheng Wetland, Jiangsu Province. *Phragmites australis* and *Spartina alterniflora* accounted for 96.73% of the diet composition of Père David's deer, while the branches and leaves of woody plants were rarely consumed, accounting for only 3.27% of the diet composition (Hua et al., 2020). To further assess the reintroduction of Père David's deer efforts, researchers also need effective information on changes in diet across seasons, including long‐term diet composition and factors that mediate dietary variation.

Hubei Shishou Milu National Nature Reserve is the habitat of the largest wild Père David's deer population in China. It is a typical lake wetland located in the middle reaches of the Yangtze River. However, due to the construction of levees after floods in 1998 and pluvial flooding in the upper reaches of the Yangtze River, the floating aquatic plants were unable to reach the reserve (Li et al., 2012; Zhang et al., 2018). In addition, following the completion of the Three Gorges Dam, the original beach embankment was isolated from natural water exchange between the Tian'E Zhou Oxbow and the Yangtze River (Ding, 2017; Zhang et al., 2018). Human activities, including planting Italian poplar and burning weeds to open up wasteland, accelerated wetland droughts. The affected hygrophytes were gradually replaced by xerophytes and mesophytes, which further accelerated wetland degradation (Li et al., 2016; Zhang et al., 2013; Zhao et al., 2010), soil erosion (Li et al., 2017), and water pollution (O'Hare et al., 2018; Wolka et al., 2018). Wetland degradation may lead to the destruction of wetland ecosystem structure (Davidson et al., 2018), biodiversity reduction (Janne et al., 2021), productivity and wetland function attenuation (Bouma et al., 2014), and other ecological environment deterioration (Fan et al., 2021). Moreover, changes to the vegetation community structure could have a subsequent impact on the largest wild Père David's deer population in China and on the food source of Père David's deer. This further affects the stability of the wetland ecosystem (Hummel et al., 2018; Motta et al., 2020).

Decreases in plant species abundance, diversity, or coverage can affect the foraging choices or habitat use of Père David's deer by changing plant–cervid interactions (Li et al., 2015, 2016). When Père David's deer population abundance increases, it results in a decrease in the diversity and abundance of plants, as seen in other deer populations in Jiangsu, China, Central Japan, and northeastern Illinois (Anderson et al., 2005; Ding, 2017; Iijima et al., 2013). With the increase in trampling frequency of Père David's deer, the soil bulk density increases, while the soil moisture content decreases (Zhou et al., 2010). Meanwhile, available phosphorus, potassium, and other soil components in the surface layers are also continuously reduced (Ding, 2017; Qian et al., 2008; Zhou et al., 2010). This results in lower plant nutrients and reduced biomass, thus preventing the growth of Père David's population within the national reserves. These current and future problems threatening the protection and management of Père David's deer in China could be addressed through the artificial planting of pasture, the implementation of habitat protection and restoration projects, human intervention in population changes (migration in and out) of the population, and other methods. Understanding the dietary composition and quantifying the nutrient composition of Père David's deer is the basis for this work. This is closely related to the type of forage to be planted, the assessment of emigration sites, and the direction of habitat restoration (Xue et al., 2022; Zhang et al., 2021).

Stable isotope analysis can provide quantitative information on the dietary contribution of various foods consumed by Père David's deer (De Smet et al., 2004). Compared with traditional methods such as behavioral observations and stomach content analyses, stable isotope analysis can provide longer‐term measurements (McCue et al., 2020). This can help us to understand variations in Père David's deer diet and provide essential information for conservation. Depending on which tissues are analyzed, the stable isotope signature would reflect food assimilation over several time scales, from a few days to the lifetime of the animal (Tieszen et al., 1983). Cell turnover in the tissue is an important factor affecting the turnover rate of different tissues in animals. Taking mammals as an example, feces would reflect food consumption of the last days, the liver for approximately the last month, the heart for the last 1–2 months, and the muscle for the last 3–4 months (Bahar et al., 2014; Caut et al., 2009; Phillips et al., 2014; Roth & Hobson, 2000; Sponheimer et al., 2006). The fur reflects food consumption during fur growth at different time scales (Ayliffe et al., 2004; Cerling et al., 2006; Schwertl et al., 2005), depending on the segment analyzed (Guo et al., 2008; O'Regan et al., 2008; Rogers et al., 2020; Schwertl et al., 2003). We used multiple deer tissues to evaluate the diet of Père David's deer according to different time scales. We also analyzed the nutrient composition of diverse plant species and combined stable isotopes and nutrient compositions to explore the relationship between the diet composition of Père David's deer and the food's nutrient content. Specifically, we aim to address the following scientific aspects of forecasting: (1) Do the stable isotope signatures of Père David's deer vary among tissue types? (2) Does the diet composition of Père David's deer change with the season? (3) Is the diet composition of Père David's deer influenced by the food's nutrient content?

## 2 MATERIALS AND METHODS

### 2.1 Study sites and sample collection

**FIGURE 1:**
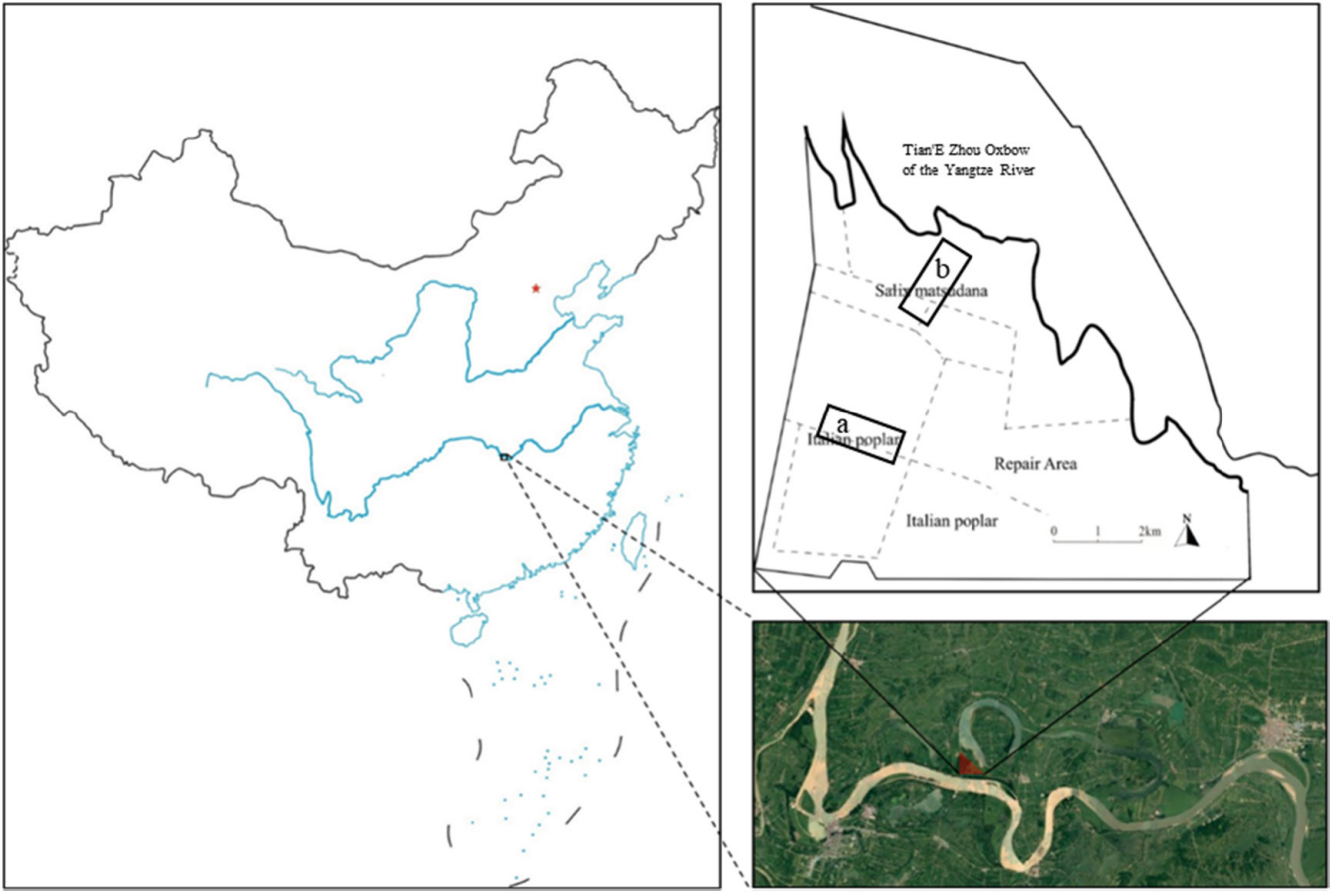
Map of the geographical location and regional distribution of the Shishou Milu National Nature Reserve in Hubei Province, China. Areas a and b are the sampling sites for plant and fecal samples.

Hubei Shishou Milu National Nature Reserve (E112°31′‐112°36′, N29°46′‐29°51′) is located in Hubei Province in China (Figure 1). With a total area of 1567 hm^2^, the reserve is adjacent to the Yangtze River to the south with the Tian'E Zhou Oxbow to the east, located at the southern end of the Jianghan Plain (Zou et al., 2013). There are approximately 72 families, 216 genera, and 321 species of wild higher plants in the reserve. The vegetation types in this area can be divided into three groups: meadow type, swamp type, and aquatic plant type. Among them, herbs are the main plant community, and there are few tree species (Zhang, Li, et al., 2019). Père David's deer is the only large mammal in the reserve.

Plant samples were collected in January 2021. The activity traces of Père David's deer (resting place traces and foraging plant traces) were investigated during our survey and combined with the observation records of the reserve staff. The main activity ranges of Père David's deer were identified as the Italian poplar forest near the oxbow and the *S. matsudana* forest near the bank of the Yangtze River (Figure 1). A 4 m^2^ sample plot was established every 150 m in areas a and b. A total of six sample plots (3 m × 2 m) were collected. In each sample plot, aboveground plant parts with signs of foraging were collected with stainless‐steel scissors in sealed bags, which were kept in ice boxes to avoid wilting of plant leaves due to temperature changes. Plants with high cover (more than one‐fifth of the sample plot) but no obvious signs of feeding were also collected.

Fresh fecal samples from the areas a and b were collected in January 2021. Père David's deer were spotted and followed by professional staff at a distance, fresh droppings were picked up along the way. One dung mound was regarded as being created by one deer. Five dung mounds were mixed into one fecal sample. A total of nine fresh fecal samples were collected. Soil and plant tissue on the surface of the feces were cleaned with brushes and tweezers and stored in sealed bags. Fecal samples were considered to show the diet in January. Fur samples were collected in April 2019 and 2020. The shedding period of Père David's deer is from March to April and August to September each year (Ding, 2017). The difference in color and size between adult and immature shedding fur masses of Père David's deer is obvious. Fur was collected through professional staff, relying on observation at a distance to ensure that one fur mass was regarded as being created by one deer. To avoid the effects of age, only the fallen fur masses from the adult Père David's deer were collected and kept in sealed bags for further processing. Approximately three to four fur masses were mixed into one fur sample, and a total of four fur samples were collected in areas a and b each year, for a total of eight fur samples collected in 2019 and 2020. The fur divided into three sections (fur tip, fur middle, and fur root) can be approximately considered to show the diet composition from October to November, December to January, and February to March (Ding, 2017; Guo et al., 2008; Schwertl et al., 2003). Muscle, liver, and heart samples were taken from one individual adult male Père David's deer that died accidentally (intraspecific struggle) on August 5, 2019, and were sliced with a sterile scalpel and stored at −80°C after being collected in sealed bags. A total of five muscle samples, five liver samples, and three heart samples were collected from this Père David's deer. Muscle samples are considered to show the diet between May and June (Caut et al., 2009; Phillips et al., 2014; Sponheimer et al., 2006). Heart samples are considered to show the diet between June and July (Bahar et al., 2014; Caut et al., 2009; Roth & Hobson, 2000). Liver samples are considered to show the diet in July (Bahar et al., 2014; Caut et al., 2009; Roth & Hobson, 2000; Sponheimer et al., 2006). Research on live animals was performed following the guidelines of the China Wildlife Protection Law, and all research protocols were approved by Hubei Shishou Milu National Nature Reserve.

### 2.2 Sample pretreatments and lipid removal

Plant samples were processed immediately upon return to the laboratory. Fresh, mature, insect‐free plant leaves were cut off with scissors and gently rinsed with distilled water. The cleaned leaves were immediately sent to a freeze dryer for drying (−40°C in a vacuum freezing dryer and 48 h of dehydration; all subsequent freeze‐drying settings were the same). The dried leaves were ground into a fine powder using a ball mill and packaged. The fecal samples were washed with distilled water and sent to a freeze dryer for drying. After being removed, the samples were ground into a fine powder using a ball mill and packaged.

The fur of Père David's deer can be roughly divided into guard fur, underfur, and intermediate fur, with guard fur being the densest and distributed throughout the body. Guard fur is approximately 40–60 mm long, and its growth rate is approximately 6–10 mm/month (Ding, 2017; Perrin & Campbell, 1980). Eight fur samples (guard fur) were picked out with tweezers and straightened, measured, and divided equally into three sections using scissors, for a total of 24 fur subsamples. After they were washed in an ultrasonic bath using a 2:1 chloroform–methanol solution to remove surface contamination and external lipids (Azorit et al., 2012), the fur subsamples were washed with distilled water and then put into an oven for drying (Hobson et al., 2000).

To further reduce the effect of lipids on stable isotopes, initially processed fur samples and tissue samples from the previous step were subjected to a 2:1 chloroform–methanol solution for further lipid extraction (Ehrich et al., 2011; Rioux et al., 2019). The mixture was shaken and stored at 4°C overnight (18 h; Folch et al., 1957). The supernatant was then removed by centrifugation for 10 min. This procedure was repeated two times (Folch et al., 1957). After three extractions, the samples were dried in an oven. After being dried for 12 h, they were washed with distilled water and then dried in a freeze dryer. After being removed, they were ground into a fine powder using a ball mill and then packaged. All test tissue samples were degreased samples.

### 2.3 Determination of plant nutrients

To further explore the effect of plant nutrients on the dietary composition of Père David's deer, freeze‐dried and ground plants were used for subsequent plant nutrient analysis. Three replicates were set up for each plant species. The mean value (±SD) of three replicates per species was used as the measurement of nutrient content.

Crude fat determination was determined according to the direct extraction method (Zou et al., 1999). Freeze‐dried plant samples (500 mg) were dissolved in 10 ml of HCl for 50 min. Then, 10 ml of ethanol was added, and the fat was extracted with 25 ml of petroleum ether, mixed, and shaken, and the supernatant was collected. The supernatant was dried (105°C for 2 h) and weighed to calculate the crude fat content (Zou et al., 1999).

The C/N and N contents were determined using an elemental analyzer (Flash EA 1112HT; Thermo Fisher Scientific) in the laboratory of the Food Inspection and Quarantine Center, Shenzhen Custom, China.

The soluble sugar content was determined according to the anthrone–H_2_SO_4_ method (Nakamura, 1968). A standard curve was established with glucose standards. Freeze‐dried plant samples (250 mg) were extracted by adding 10 ml of distilled water in boiling water for 30 min, and the supernatant was mixed with ethyl anthranilate‐concentrated H_2_SO_4_, shaken, and held in a boiling water bath for 1 min. The absorbance was measured at 630 nm by UV spectrophotometry (Nakamura, 1968).

The crude protein content was determined by the Coomassie brilliant blue method (Hayes, 2020). A standard curve was established with bovine serum protein standards. Freeze‐dried plant samples (100 mg) were weighed and extracted with distilled water for 2 h at room temperature, the supernatant was mixed with Kaumas Brilliant Blue G250, and the absorbance was measured at 595 nm with UV spectrophotometry (Hayes, 2020).

Condensed tannin (proanthocyanidins) was determined by the acid butanol method (Wei et al., 2014). Freeze‐dried plant samples (50 mg) were dissolved in 1 ml of methanol and mixed with 6 ml of 95% butan‐1‐ol and 5% concentrated HCl in 10 ml test tubes. The tubes were sealed and placed in a water bath at 95°C for 1 h. The color was observed after cooling to room temperature. The alcoholysis under acidic conditions converts the extended units of condensed tannins into colored anthocyanins. The darker the color, the higher the tannin concentration (Wei et al., 2014).

### 2.4 Stable isotope analyses

After the pretreatments, the carbon and nitrogen stable isotope ratios of the samples were analyzed using an elemental analyzer (Flash EA 1112HT; Thermo Fisher Scientific) coupled with an isotope ratio mass spectrometer (Delta V Advantage; Thermo Fisher Scientific) in the laboratory of the Food Inspection and Quarantine Center, Shenzhen Custom, China. Approximately 0.200 mg (±0.001 mg) of sample plant and tissues was weighed and inserted into 4 mm × 6 mm tin cups. The carbon and nitrogen stable isotope ratios of the sample were expressed in delta notation (difference between sample and standard) as parts per thousand (‰):

$$\delta =\left[\left(R_{\text{sample}}/R_{\text{standard}}\right)-1\right]\times 1000.$$

where *R* is the abundance ratio of heavy isotopes to light isotopes in the sample, ^13^C/^12^C and ^15^ N/^14^ N. *R*_sample_ is the measured isotope ratio; *R*_standard_ is the isotope ratio of reference materials. All results are reported relative to atmospheric nitrogen as the standard for δ^15^N and to Cretaceous belemnite (Belemnitella Americana) from the Peedee Formation of South Carolina for δ^13^C. Laboratory standards (glycine and urea) were run every 12 samples to correct any instances of instrument drift. The analytical precision was ±0.1‰ for ^13^C and ±0.2‰ for ^15^N.

### 2.5 Mixing models and statistical analyses

For animals with two or more food sources, the proportion of food in animal diets can be determined according to the isotopic mass balance equation:

$$\delta^{13}C_{i}=\sum_{j=1}^{n}\left[f_{\mathrm{ij}}^{\prime }\left(\delta^{13}C_{i}+\mathrm{\Delta C}\right)\right]$$

$$\delta^{15}N_{i}=\sum_{j=1}^{n}\left[f_{\mathrm{ij}}^{\prime }\left(\delta^{15}N_{i}+\mathrm{\Delta N}\right)\right]$$

$$\sum_{j=1}^{n}f_{\mathrm{ij}}^{\prime }=1.$$

where δ^13^C_i_ and δ^15^N_i_ represent the carbon and nitrogen isotopic compositions of consumers, respectively; δ^13^C_j_ and δ^15^N_j_ represent the carbon and nitrogen isotopic compositions of possible foods, respectively; ΔC and ∆N represent the carbon and nitrogen isotope discrimination values of nutrient grade, respectively; and $f_{\mathrm{ij}}^{\prime }$ is the proportion of food in the consumers' food sources (Parnell et al., 2013; Saito et al., 2001).

To estimate the relative contribution of multiple food sources to Père David's deer diet, the Bayesian isotope mixing model SIMMr (stable isotope mixing models) in R (Jackson et al., 2008; Parnell et al., 2010) was used, as it provides advantages over standard, mass balance multisource mixing models (Phillips & Gregg, 2003). SIMMr can integrate sources of variability associated with multiple sources, trophic enrichment factors, and isotopic values, and its outputs represent true probability density functions rather than a range of feasible solutions (Moore & Semmens, 2008; Parnell et al., 2013; Phillips et al., 2014).

As different tissue types metabolize isotopes at different speeds, before applying a mixing model, these systematic differences must be corrected (Phillips et al., 2014). To more accurately reflect the contribution of each food source to Père David's deer, trophic enrichment factors (TEFs) were used to correct for the enrichment of stable isotope signatures between the tissue and diet of Père David's deer. Since TEF values for various tissues of Père David's deer are not available, the TEFs from the currently published literature were used instead. We are aware that incorrect use of TEF can produce erroneous determinations of food sources (Bond & Diamond, 2011). Estimated TEFs reached 2.31 ± 0.20‰ for ∆^13^C (Caut et al., 2009) and 4.86 ± 0.94‰ for ∆^15^ N for fur (Rioux et al., 2020); 1.48 ± 0.14‰ for ∆^13^C (Caut et al., 2009) and 3.02 ± 0.12‰ for ∆^15^ N (Caut et al., 2009) for muscle; 0.36 ± 1.86‰ for ∆^13^C (Stephens et al., 2022) and 3.55 ± 0.05‰ for ∆^15^ N (Caut et al., 2009) for liver; 2.92 ± 0.01‰ for ∆^13^C (Caut et al., 2009) and 2.95 ± 0.01‰ (Caut et al., 2009) for ∆^15^ N for heart; and −0.9 ± 0.2‰ (Sponheimer et al., 2006) for ∆^13^C and 1.9 ± 0.3‰ for ∆^15^ N (Steele & Daniel, 1978) for feces. Due to sampling time constraints, the plant samples collected in January 2021 were used as a potential food source for all mixing model analyses. Sixteen species of plants were collected and grouped into four groups according to the stable isotope signature similarity, photosynthetic pathway (Phillips et al., 2014), ANOVA testing, and feeding preferences of Père David's deer (Ding, 2017; Zhang, Fu, et al., 2019; Zhang & Yang, 2017). The four groups were C_3_ forbs (C_3_‐F, *n* = 10), C_3_ grasses (C_3_‐G, *n* = 2), C_4_ grasses (C_4_‐G, *n* = 2), and Other (avoided, *n* = 2), respectively. The negatively correlated source proportions were combined to gain precision in the calculated proportions (Parnell et al., 2013; Phillips et al., 2014). Therefore, C_3_‐F, C_3_‐G, and C_4_‐G were included in the Bayesian isotope mixing model. Estimates are reported with their 95% credible intervals, and the results are displayed as median, 5% and 95% percentile values (Parnell et al., 2013).

One‐way analysis of variance (ANOVA) followed by Tukey's post hoc test was used to test differences in stable isotope values among the potential food sources (C_3_‐F, C_3_‐G, and C_4_‐G) and fur subsamples. Then, a two‐way ANOVA was conducted to evaluate the effects of fur subsamples (fur tip, fur middle, and fur root) and sampling time (2019 and 2020) on the stable isotope values of fur. The coefficient of variation (COV = SD/mean) was used to describe the degree of variation in the δ^13^C and δ^15^N values of different tissue samples. Pearson correlation analysis was performed to determine the relationship between the nutrient composition of each food source and its proportional contribution to the diet of Père David's deer. The values of nutrients were determined individually for each plant in the four groups (C_3_‐F, C_3_‐G, C_4_‐G, and Other). The values of the proportional contribution to the diet were determined by the mean of the Bayesian mixture model of the three potential food sources (C_3_‐F, C_3_‐G, and C_4_‐G) in the selected samples, and the proportional contribution to the diet for plants (Other) not consumed by Père David's deer was 0. To reduce the error caused by the difference in season and year, three samples (Collected Fur Middle in 2019 and 2020, Feces) were selected for analysis in which the response dietary time window overlapped with the time of plant collection. The Pearson correlation coefficient (*r*) and *p‐*values were determined using a two‐tailed Pearson correlation analysis. All tests were two‐tailed, and the acceptable significance level was α = 0.05. The results are reported as the mean ± 1 SD unless otherwise stated. All statistical analyses were performed using SPSS 19.0 (SPSS for Windows; SPSS, Inc.).

## 3 RESULTS

### 3.1 Stable isotope values of potential food sources and samples of Père David's deer

A total of 16 species of plants were collected in this survey, 14 of which were potential food sources for Père David's deer (Table 1). *Arundo donax* and *Polygonum perfoliatum* (Other) have been studied to confirm that they are not consumed by Père David's deer (Ding, 2017; Zhang, Fu, et al., 2019; Zhang & Yang, 2017). One‐way ANOVA showed that the δ^13^C and δ^15^N values of the three types (C_3_‐F, C_3_‐G, and C_4_‐G) of plants were significantly different (δ^13^C, df = 2/45, *F* = 1189.73, *p* < .001; δ^15^N, df = 2/45, *F* = 4.86, *p* = .012). Tukey's post hoc test showed that the difference in δ^15^N values between C_3_‐G and C_4_‐G was not significant (df = 1/41, *F* = 0.06, *p* = .807). Other group of *Arundo donax* had the lowest δ^13^C value of −27.93 ± 0.47‰. Based on the one‐way ANOVA of data from different sections in the same year, there was a significant difference in the δ^15^N value of fur subsamples in different sections in 2019 (df = 2/9, *F* = 0.43, *p* = .043), and a significant difference in the δ^13^C value of fur subsamples in different sections in 2020 (df = 2/9, *F* = 258.77, *p* < .001). Two‐way ANOVA revealed that the fur of different sections had no significant differences in δ^13^C and δ^15^N values (Table 2). Sampling time (2019 and 2020) only significantly influenced the δ^15^N values of the fur. The interactions between sampling time and section significantly affected the δ^15^N values of fur (Table 2).

**TABLE 1:** Classification, carbon, and nitrogen stable isotope ratios of plants in Shishou Milu National Nature Reserve

Type	Family	Species	Number	δ^13^C (‰)	δ^15^N (‰)	Degree of appetite
C_3_‐F	Umbelliferae	*Daucus carota*	3	−31.84 ± 0.64	−0.70 ± 0.84	++
	Umbelliferae	*Apium graveolens*	3	−31.98 ± 0.02	0.96 ± 0.04	+
	Polygonaceae	*Polygonum lapathifolium*	3	−29.56 ± 0.20	−2.99 ± 1.07	++
	Labiatae	*Leonurus artemisia*	3	−32.65 ± 0.32	−1.31 ± 0.35	+
	Asteraceae	*Hemistepta lyrata*	6	−31.88 ± 0.62	−0.55 ± 0.92	+
	Geraniaceae	*Geranium carolinianum*	3	−30.98 ± 0.23	−1.15 ± 0.04	+
	Plantaginaceae	*Plantago asiatica*	3	−31.92 ± 0.27	−3.09 ± 0.24	++
	Leguminosae	*Trifolium repens*	3	−31.85 ± 0.61	−1.96 ± 0.69	++
	Brassicaceae	*Capsella bursa‐pastoris*	3	−30.17 ± 0.79	3.03 ± 4.38	+
	Cyperaceae	*Carex* spp.	3	−31.67 ± 0.33	1.40 ± 3.03	+
			33	−33.08 ± 1.48^a^	2.58 ± 2.69^b^	
C_3_‐G	Gramineae	*Lolium perenne*	3	−32.29 ± 0.13	3.64 ± 2.70	+
	Gramineae	*Roegneria kamoji*	3	−34.26 ± 2.02	0.98 ± 2.41	+
			6	−31.32 ± 0.97^b^	−0.67 ± 2.54^a^	
C_4_‐G	Gramineae	*Cynodon dactylon*	6	−14.47 ± 0.37	0.01 ± 0.47	++
	Gramineae	*Phragmites australis*	3	−13.12 ± 0.26	−1.70 ± 0.52	++
			9	−14.24 ± 0.68^c^	−0.99 ± 1.49^a^	
Other	Gramineae	*Arundo donax*	3	−27.93 ± 0.47	−3.06 ± 0.81	−
	Polygonaceae	*Polygonum perfoliatum*	3	−31.63 ± 0.35	−3.72 ± 0.67	−

*Note*: Type: C_3_‐F (C_3_ forbs), C_3_‐G (C_3_ grasses), C_4_‐G (C_4_ grasses), and Other (avoided). The last line of each type is the data after the combination of that type and different letters denote significant differences at *p* < .05. “+” and “−” indicate the food preference of Père David's deer (Ding, 2017; Zhang, Fu, et al., 2019; Zhang & Yang, 2017).

**TABLE 2:** A summary of two‐way ANOVA for δ^13^C and δ^15^N values of fur divided into three sections (fur tip, fur middle, and fur root) and sampling time (2019 and 2020) were selected as treatments

Variable	df	δ^13^C		δ^15^N	
		*F*	*p*	*F*	*p*
Section	2	2.713	.093	1.350	.284
Sampling time	1	0.080	.780	213.938	.000
Section × Sampling time	2	1.160	.336	6.189	.000

**FIGURE 2:**
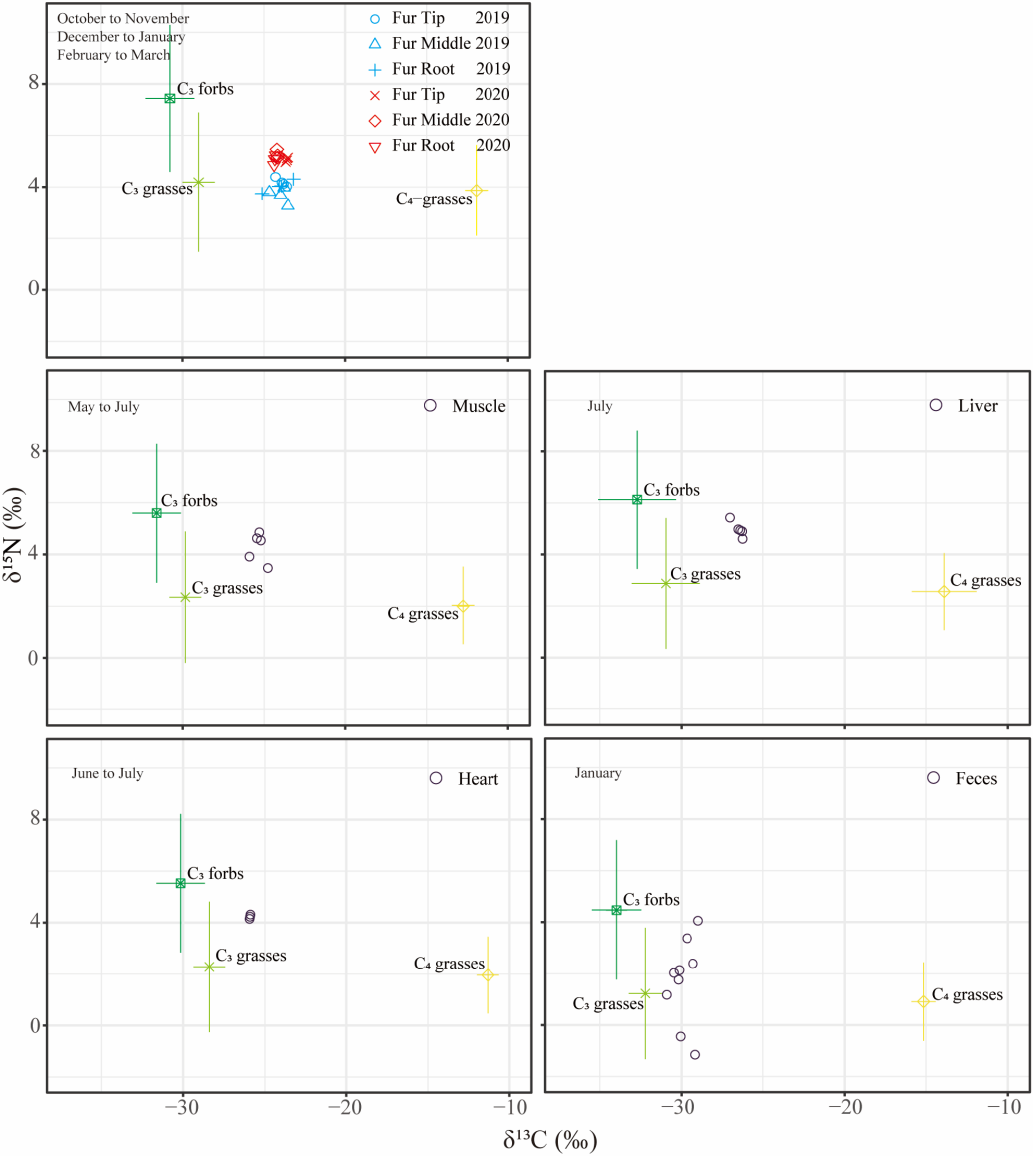
Stable isotope signatures of carbon and nitrogen from potential food sources (solid points and error bars, corrected for TEF) and raw data from consumers (fur, muscle, liver, heart, and feces of Père David's deer) in scatter plots. The upper right corner indicates the diet window for the sample response.

Figure 2 shows the relationship between different tissues and potential food sources. The muscle, liver, and heart samples were collected from the same Père David's deer. The δ^13^C values of fur, feces, muscle, liver, and heart from Père David's deer were close to those of C_3_ plants (Figure 2). We analyzed the stable isotope signatures of different tissues and found that the stable isotope signatures of Père David's deer vary among tissue types. We observed the lowest enrichment in the feces (δ^13^C: −29.87 ± 0.64‰, δ^15^N: 1.70 ± 1.7‰), followed by the liver (δ^13^C: −26.48 ± 0.31‰, δ^15^N: 4.97 ± 0.30‰). This is the same as our predictions for the fecal reflect the diet of the last days, the liver for approximately the last month, and other tissues reflect a longer time window (Bahar et al., 2014; Caut et al., 2009; Phillips et al., 2014; Roth & Hobson, 2000, Sponheimer et al., 2006). The deviations of δ^13^C values of each tissue were small (all COV < 0.05). The deviations of δ^15^N values of muscle, liver, and heart were similar (all COV < 0.1). Although feces and fur were collected from many different Père David's deer, the deviations in the δ^15^N values of feces (COV = 0.98) were larger than those of fur (all COV < 0.1).

### 3.2 Diet composition estimates of Père David's deer

The results of the Bayesian mixture model indicated that the C_3_ plants (including C_3_‐G and C_3_‐F) were the main diet component for Père David's deer in Hubei Shishou Milu National Nature Reserve. However, there were differences in the contribution of food sources reflected by different tissues of Père David's deer.

**FIGURE 3:**
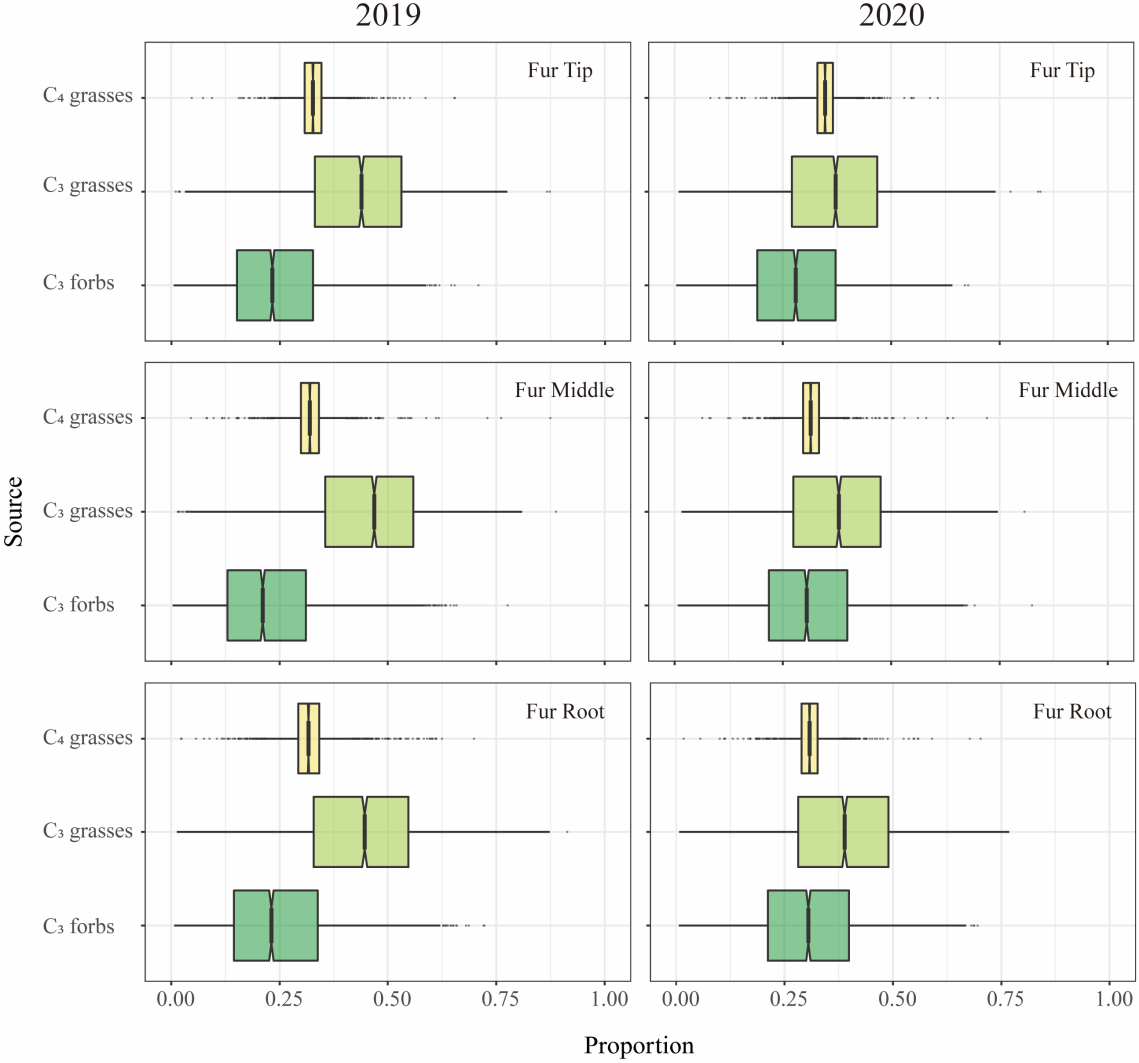
Proportional contribution of potential food sources (50%, 75%, and 95% confidence intervals) to the diet of Père David's deer in different years (2019 and 2020) using segmented fur (tip, middle, and root) and estimated using stable isotopic mixing models.

The segmented fur samples indicated the composition of the diet at different times. The segmented fur samples (fur tip, fur middle, and fur root) for 2019 indicated that Père David's deer consumed mainly C_3_‐G (42.7% [11.9–65.3], 45.2% [12.2–68.0], and 42.8% [9.5–68.0]), followed by C_4_‐G (32.6% [25.6–.6], 31.9% [24.8–40.2], and 32.0% [22.3–43.4]), and C_3_‐F (24.7% [4.7–.4], 22.9% [3.5–52.9], and 25.2% [4.5–55.9]), respectively (Figure 3). The segmented fur samples for 2020 showed the same trend as those in 2019. Père David's deer consumed mainly C_3_‐G (36.5% [8.9–62.1], 37.3% [9.9–64.0], and 38.0% [8.7–65.5]), as well as C_4_‐G (34.8% [28.2–41.8], 31.6% [25.6–38.1], and 30.9% [24.1–38.1]) and C_3_‐F (28.7% [6.2–53.6], 31.1% [7.1–56.4], and 31.1% [6.7–57.7]), respectively (Figure 3).

**FIGURE 4:**
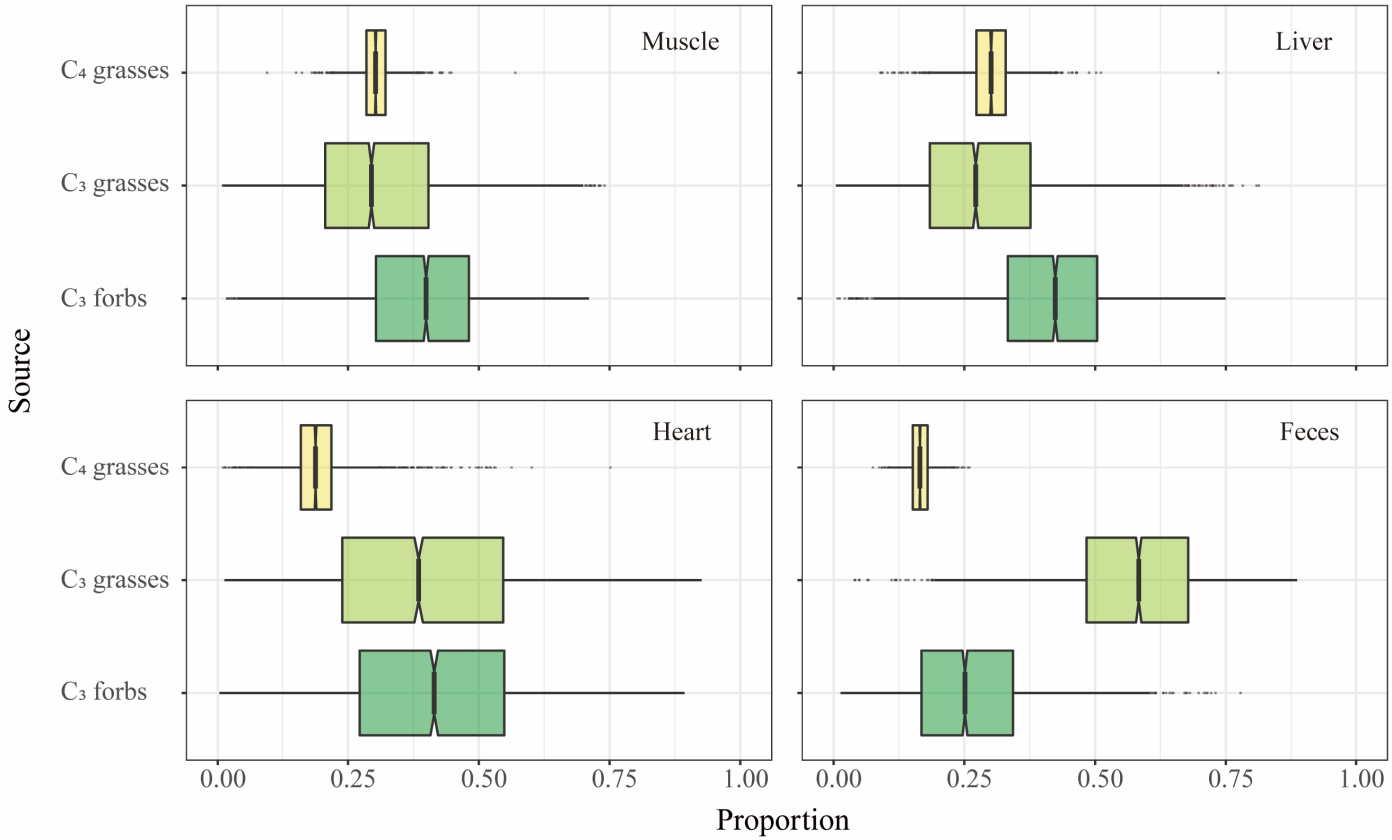
Proportional contribution of potential food sources (50%, 75%, and 95% confidence intervals) to the diet of Père David's deer using different tissue (muscle, liver, heart, and feces) and estimated using stable isotopic mixing models.

The muscle, liver, and heart samples also showed the composition of the summer diet of this unexpectedly dead Père David's deer. Among them, muscle samples showed that the Père David's deer consumed mainly C_3_‐F (39.0% [10.3–62.0]) but also C_3_‐G (30.6% [6.0–62.1]) and C_4_‐G (30.4% [24.9–36.1]) between May and June (Figure 4). Heart samples showed that Père David's deer consumed mainly C_3_‐F (41.5% [7.4–73]), followed by C_3_‐G (39.2% [6.0–78.1]) and C_4_‐G (19.3% [9.3–32.8]) between June and July (Figure 4). Liver samples showed that the Père David's deer consumed mainly C_3_‐F (41.6% [14.1–63.2]), followed by C_4_‐G (30.3% [21.0–38.7]) and C_3_‐G (28.1% [5.5–59.6]) in July (Figure 4).

Fecal samples represent dietary composition over the previous days and present more individual variability (Phillips et al., 2014). We found that Père David's deer consumed mainly C_3_‐G (57.2% [26.2–80.4]) in January, and the contribution of C_3_‐G in fecal samples was much higher than that in other samples, followed by C_3_‐F (26.3% [5.3–54.3]) and C_4_‐G (16.5% [12.4–21.0]) (Figure 4).

### 3.3 Nutrient content of the potential food source

The contents of various nutrients from these 16 representative plants are listed in Table 3. The moisture content of different plants ranged from 70% to 80%. The difference between *C. dactylon* with the highest crude fat content and *P. australis* with the lowest crude fat content of grasses was 0.8%. The range of crude fat content of forbs was larger than that of grasses, from 2.5% to 6.6%. The C/N ratios of *C. dactylon* and *P. asiatica* were 20.09 and 18.54, respectively, which were higher than those of other plants in the range of 9%–13%. The nitrogen content of C_3_ plants was higher than that of C_4_ plants, but the C/N ratio of *T. repens* with the highest nitrogen content was the lowest. There were differences in soluble sugar content and crude protein content among different plant types (soluble sugar: df = 3/44, *F* = 6.18, *p* = .010; crude protein: df = 3/44, *F* = 11.93, *p* < .001). Other (avoided) group of plants (*Arundo donax* and *Polygonum perfoliatum*) had the lowest soluble sugar content. Only *P. lapathifolium* and *Apium graveolens* contained condensed tannins, and the condensed tannins of *P. lapathifolium* were higher than those of *A. graveolens*.

**TABLE 3:** Nutrient content of plant in Shishou Milu National Nature Reserve

Type	Plant	Moisture content (%)	Crude fat content (%)	C/N	N content (%)	Soluble sugar (%)	Crude protein (%)	Condensed tannins
C_3_‐G	*Lolium perenne*	74.58 ± 0.50	4.60 ± 0.05	10.92 ± 2.42	4.12 ± 1.15	3.47 ± 0.20	11.87 ± 0.25	−
	*Roegneria kamoji*	73.94 ± 0.29	5.10 ± 0.69	14.01 ± 1.45	3.53 ± 1.37	2.19 ± 1.23	9.47 ± 0.69	−
C_3_‐F	*Daucus carota*	80.60 ± 0.11	6.61 ± 0.21	11.88 ± 2.23	3.55 ± 0.71	1.53 ± 0.76	6.92 ± 0.94	−
	*Apium graveolens*	80.67 ± 0.10	5.17 ± 0.87	11.72 ± 0.25	2.95 ± 0.37	2.16 ± 0.20	4.59 ± 0.23	+
	*Polygonum lapathifolium*	80.34 ± 0.54	4.83 ± 0.38	13.07 ± 0.12	3.48 ± 0.08	2.41 ± 0.21	9.16 ± 0.87	++
	*Leonurus artemisia*	77.39 ± 0.73	5.21 ± 0.76	12.42 ± 0.63	2.98 ± 0.54	1.52 ± 0.29	5.36 ± 0.31	−
	*Hemistepta lyrata*	78.75 ± 0.14	3.76 ± 0.97	12.44 ± 0.82	3.43 ± 0.57	2.49 ± 0.48	11.65 ± 0.55	−
	*Geranium carolinianum*	73.59 ± 0.02	2.68 ± 0.54	13.14 ± 0.11	3.38 ± 0.03	1.76 ± 0.47	4.92 ± 0.44	−
	*Plantago asiatica*	81.38 ± 0.97	3.92 ± 0.59	18.54 ± 0.08	2.43 ± 0.06	1.39 ± 0.36	10.41 ± 0.76	−
	*Trifolium repens*	79.78 ± 0.08	3.46 ± 0.23	9.20 ± 1.13	5.49 ± 1.46	1.43 ± 1.13	8.33 ± 0.02	−
	*Capsella bursa‐pastoris*	76.00 ± 0.36	3.79 ± 0.12	11.29 ± 1.00	3.57 ± 0.19	2.13 ± 1.78	8.84 ± 0.61	−
	*Carex* spp.	70.35 ± 0.90	2.56 ± 0.29	11.04 ± 0.69	3.90 ± 0.21	2.35 ± 0.28	8.85 ± 0.16	−
C_4_‐G	*Cynodon dactylon*	71.25 ± 0.79	5.21 ± 0.59	20.09 ± 0.32	2.28 ± 0.21	3.28 ± 0.85	7.69 ± 0.56	−
	*Phragmites australis*	72.57 ± 0.54	4.41 ± 0.74	13.87 ± 0.16	2.88 ± 0.07	2.24 ± 0.62	8.97 ± 0.69	−
Other	*Arundo donax*	73.76 ± 0.27	5.04 ± 0.34	11.20 ± 0.12	3.92 ± 0.05	1.09 ± 0.25	3.57 ± 0.09	−
	*Polygonum perfoliatum*	77.81 ± 0.24	4.57 ± 0.09	12.82 ± 0.18	3.21 ± 0.29	1.37 ± 0.33	4.26 ± 0.30	−

*Note*: Type: C_3_‐G (C_3_ grasses), C_3_‐F (C_3_ forbs), C_4_‐G (C_4_ grasses), and Other (nonedible). “+” and “−” indicate the presence or absence of condensed tannins.

The Pearson correlation test was used to analyze the correlation between the nutrient composition of each food source and its proportional contribution to the diet of Père David's deer (Table 4). In the three samples selected, soluble sugar and crude protein were correlated (soluble sugar: df = 14, *r* = .695, *t* = 3.62, *p* = .003; df = 14, *r* = .543, *t* = 2.42, *p* = .030; df = 14, *r* = .501, *t* = 2.17, *p* = .048 in order; crude protein: df = 14, *r* = .666, *t* = 3.34, *p* = .005; df = 14, *r* = .636, *t* = 3.09, *p* = .008; df = 14, *r* = .622, *t* = 2.97, *p* = .010 in order) with the proportional contribution of the four source groups (C_3_‐G, C_3_‐F, C_4_‐G, and Other) to the diet.

**TABLE 4:** Correlations between nutrient composition of each food source and its proportional contribution (C_3_‐G [mean], C_3_‐F [mean], C_4_‐G [mean], and Other [0]) to the diet of Père David's deer by Pearson correlation analysis

	Selected sample	Moisture content/%	Crude fat content/%	C/N	N content/%	Soluble sugar/%	Crude protein/%
Proportional contributions of each dietary source	Fur Middle 2019	−0.226	0.044	0.186	−0.030	0.695*	0.666*
	Fur Middle 2020	0.013	−0.102	0.125	−0.030	0.543*	0.636*
	Feces	−0.014	−0.005	−0.094	0.200	0.501*	0.622*

* Indicate a significant correlation at the 0.05 levels (two‐sided).

## 4 DISCUSSION

### 4.1 Diet composition of Père David's deer

Research on the feeding habits of ungulates is essential to understanding the interaction between wild animals and the environment. It is also the basis for evaluating population ecology issues such as habitat quality, environmental tolerance, and feeding strategies of wild animals (Zhang et al., 2019). We measured diet composition in multiple tissues, and observed differences in diet composition among feces, fur, and other tissues, which will be useful for determining variations in the diet composition of Père David's deer. We observed that the autumn and winter diets estimated by fur samples indicated a diet dominated by C_3_‐G (C_3_ grasses, 42.7%–57.2%, mean), and the lowest proportional contribution of C_3_‐F (C_3_ forbs, 22.9%–31.1%, mean) to the diet. The summer diet estimated by muscle and liver samples showed that the C_3_‐F (30.9%–41.6%, mean) was the main plant type consumed by Père David's deer. In the one‐way ANOVA and two‐way ANOVA of fur subsamples, we found that the results of the effect of the factor sectioning on the stable isotope signature of the fur subsamples were not the same. However, combined with the analysis of the Bayesian mixture model, we believe there is little variation in the composition of the winter diet of Père David's deer. We speculated that the reason for the lack of significant change is that the diet of the Père David's deer was similar during this time. Alternatively, for Père David's deer, fur may have been integrated into the average diet during the entire fur growth (Rogers et al., 2020).

Comparing tissues that respond to dietary information on different time scales could provide more complex response information on food selection (Rogers et al., 2020). We observed that C_3_‐G was consumed much more from November to January (compared with other months in SIMMr with a probability of approximately 0.41). This is consistent with the results from the fecal samples (collected in January), which demonstrated greater C_3_‐G consumption. Careful analysis of the food consumed by Père David's deer revealed a larger range of proportional contributions from G_3_‐G and C_3_‐F. We speculate that this is related to the fact that the Père David's deer consumed more C_3_ plants than C_4_ plants. The deviations of δ^15^N values of feces (COV = 0.98) were larger than those of fur (COV < 0.1). Fecal samples represent the dietary composition over the previous days and present more individual variability. This indicated that the day‐to‐day diet is variable, but relatively consistent when considered over several months (Meng et al., 2010; Wang & Wang, 2011). Muscle, heart, and liver samples were all from the same Père David's deer, which affects our prediction of the summer diet composition. However, considering the little variation in habitats (Zhang, Fu, et al., 2019; Zou et al., 2013) and that Père David's deer are gregarious (Ding, 2017), our results can still provide information on the summer diet composition of Père David's deer. Smaller sample sizes may also affect the analysis of diets (Phillips et al., 2014), but we can still reveal dietary trends throughout the seasons in endangered species by using several body tissues that have different turnover time.

Similar to Wang and Wang (2011), who found that Père David's deer consumed more C_3_‐F plants in spring and summer, we also found that C_3_‐F became the main food consumed from May to July. This change is the opposite of the autumn and winter months (October to March). We speculate that these changes may be caused by differences in species richness due to seasonal variations in resources (Seto et al., 2015; Taillon et al., 2006). C_3_‐F biomass is higher in spring and summer and lower in autumn and winter, while graminoids (C_3_‐G and C_4_‐G) remain higher in autumn and winter (Zhang, Li, et al., 2019). This change in diet, brought about by changes in biomass, is more evident in the reintroduction of Père David's deer in the Jiangsu wetland in China. In the coastal wetland of Jiangsu Dafeng Milu National Nature Reserve, exotic *S. alterniflora* has invaded and become the dominant species (Bao & Shi, 2007; Wang et al., 2006; Zhang et al., 2008). Consequently, *S. alterniflora* gradually became the main food source of Père David's deer, which resulted in a relatively narrow selection of diet composition (Ding, 2009; Ji et al., 2011; Zhang, 2015; Zhao et al., 2010). When the preferred high‐quality food resources are limited, ungulates are forced to use low‐quality food resources (Gebert & Verheyden‐Tixier, 2001; Miranda et al., 2012). This change in feeding behavior is also consistent with other Cervidae (Dumont et al., 2005; Johnson et al., 2001; Zhang et al., 2020).

Our results were different from the diet composition of Père David's deer in northern China (mainly in Jiangsu Dafeng Milu National Nature Reserve and Beijing Milu Ecological Research Center), where C_4_ plants were the main food source (70.36%) (Wang & Wang, 2011; Zhang, 2015). Although the change in habitat shows a change in the diet composition of Père David's deer, gramineous plants remain the main food source of Père David's deer (Ding, 2009; Wang & Wang, 2011; Zhang, 2015). We believe this difference in diet is due to differences in habitat types. The geographical location, climate, and other ecological factors of coastal wetlands (Jiangsu Dafeng Milu National Nature Reserve) and lake wetlands (Hubei Shishou Milu National Nature Reserve) influence the diet. The changes in the diet brought about by habitat change prove that the temporal and spatial patterns of plant species and their abundance may be a key factor in determining the diet composition. A study on the correlation between feeding habits of *Cervus nippon* and geographical location also supported this inference (Takatsuki, 2009). *C. nippon* mainly feed on *Sasa nipponica* and other graminoids in southern Hokkaido (northern Japan), while they mainly feed on browse and fruits such as *Aucuba japonica*, *Eurya japonica*, and acorns of *Lithocarpus edulis* in southern Japan (Campos‐Arceiz & Takatsuki, 2005; Endo et al., 2017; Takatsuki, 2009). We also observed that Père David's deer were mixed feeders, similar to most Cervidae (Meng et al., 2010), despite differences in the diet composition of Père David's deer and other Cervidae (Zhong et al., 2019). For example, *Cervus nippon* and *Cervus elaphus* are also typically mixed feeders (Zhong et al., 2019), with trees and shrubs comprising their main food sources (Cui et al., 2007; Gebert & Verheyden‐Tixier, 2001; Krojerová‐Prokešová et al., 2010). Thus, we suggest that the availability of food items is an important factor influencing diet composition of Père David's deer. In turn, habitat variation affects the availability of food items (Zhong et al., 2019). This provides direction for the reintroduction of Père David's deer as a targeted habitat restoration and artificial intervention in their diet (Beest et al., 2010).

### 4.2 Effects of nutrient content on diet selection of Père David's deer

The knowledge accumulated in the field of nutritional ecology shows with increasing clarity that animal metabolism and diet selection are associated with synergistic and antagonistic assimilation strategies (Felton et al., 2016). We observed that the crude protein and soluble sugars of plants showed a correlation with their proportional contribution to the diet (Table 4). The results indicated that Père David's deer selectively fed on plants with high protein and soluble sugar contents in autumn and winter. The energy and nutrients of food are absorbed and distributed to various physiologic functions (Boggs, 2009). Protein is one of the most important nutrients for ungulates (Smith, 1978). For most ungulate herbivores, obtaining adequate protein is an important factor affecting diet selection in winter (Berteaux et al., 1998; Demment & Soest, 1985; Illius & Gordon, 1990; Jarman, 1974; Workman & Schmitt, 2012). A study showed that to maintain the metabolic requirements of *Odocoileus virginianus* in winter, the crude protein content in the diet should be within 13%–16% of the dietary intake (Soest, 2018). In winter, the mean crude protein content of the 14 plant species mainly consumed by Père David's deer in Shishou was 8.36% (Table 3). The crude protein content in the diet of *C. elaphus* in Europe reaches 5.7% and 5.0% to ensure their protein requirements (Maloiy et al., 1970; Verheyden‐Tixier et al., 2008; Yousef Elahi & Rouzbehan, 2008). This suggests that Père David's deer in Shishou can obtain protein to ensure basic needs in winter. Soluble sugars are also an important energy source for ungulates (Zhang, 2015). Soluble sugars increase rumen fluid volume and dilution rate (Schingoethe et al., 1980; Windschitl & Stern, 1988). It also promotes the absorption and utilization of protein in ruminant (Beever et al., 1978). A study by Verheyden‐Tixier et al. (2008) on selection of nutrients in red deer indicated that soluble sugar content was an important factor influencing diet selection and was more important than protein content. This is similar to the results of Hobbs et al. (1983), who compared the nutritional ecology of montane ungulates during winter, that ungulates have a strong preference for plants rich in digestible soluble sugars. Our result also is consistent with the diet selection tendency of Père David's deer in other reserves (Jiangsu Dafeng Milu National Nature Reserve and Beijing Milu Ecological Research Center; Wang & Wang, 2011; Zhang, 2015).

Our studies indicated the presence of plants with high condensed tannin levels in the diet of Père David's deer, such as *P. lapathifolium* and *Apium graveolens*, and some studies have found that Père David's deer are fond of eating *P. lapathifolium* (Ding, 2017; Zhang, Fu, et al., 2019; Zhang & Yang, 2017). Tannin, as a typical plant secondary metabolite, has attracted extensive attention. Tannins have the ability to bind and precipitate proteins, which affects the protein and nitrogen retention rates of ungulates (Estell, 2010; Qiu, 2016). Tannins can combine with carbohydrates to a certain extent and affect the nutrient absorption of food by ungulates (Mueller‐Harvey, 2006). The intake of tannins by Père David's deer is similar to that by other Cervidae. Bergvall and Balogh (2009) and Bergvall and Leimar (2005) showed *Dama dama* consumed high‐tannin food even in the presence of a low‐tannin option. The behavior confirmed that the Cervidae could keep the plant's secondary metabolites at safe levels by adjusting feeding patterns and their bioinvertase system (Chapman et al., 2010; Champagne et al., 2020; Sorensen et al., 2005; Verheyden‐Tixier & Duncan, 2000). These results indicate that tannins affect diet selection, but have little effect on Cervidae diets in natural environments (Champagne et al., 2020).

## 5 CONCLUSIONS

Our results showed that the autumn and winter diets estimated by fur and fecal samples indicated a diet dominated by C_3_ grasses (42.7%–57.2%, mean), and the summer diet estimated by muscle and liver samples was dominated by C_3_ forbs (30.9%–41.6%, mean). The Pearson correlation test indicated that crude protein and soluble sugars were important factors influencing the winter diet selection of Père David's deer to some extent. The results obtained therefore suggest the existence of seasonal dietary variation and possible targeted selection for nutrients in Père David's deer. The use of different samples combined with overlapping time periods responds to the dietary variation, which to some extent solves the problem of analytical precision caused by the difficulty of sampling endangered animals. The rich plant resources of the Tian'E Zhou Lake wetland provide a good foundation for the development of the Père David's deer population, but the increase in potential food sources also provides a challenge for the accurate analysis of stable isotopes. We suggest improving the precision of stable isotope analysis by combining different dietary analysis methods to further differentiate and a priori potential food sources. Additionally, to determine the nutritional strategy, a long‐term follow‐up survey of individual Pere David deer is recommended. The observation of diet structure and selection for nutrients is the basis for assessing population development or establishing long‐term effective conservation measures for endangered species. Comprehensively evaluating the diet selection mechanisms for Père David's deer and carefully maintaining the reserve should be highly supported, ongoing goals.

## AUTHOR CONTRIBUTIONS

**Hao‐Lin Wang:** Conceptualization (supporting); data curation (equal); formal analysis (lead); investigation (equal); methodology (equal); software (lead); visualization (equal); writing – original draft (lead); writing – review and editing (supporting). **Yue Zhao:** Data curation (supporting); investigation (equal); methodology (supporting); writing – original draft (supporting). **Fei‐Jie Wang:** Conceptualization (supporting); formal analysis (supporting); investigation (equal); methodology (supporting); software (supporting). **Xin‐Jia Sun:** Conceptualization (supporting); formal analysis (supporting); investigation (equal); methodology (supporting); software (supporting). **Jian‐Qiang Zhu:** Conceptualization (supporting); funding acquisition (supporting); methodology (supporting); resources (equal); writing – review and editing (supporting). **Yu‐Ming Zhang:** Funding acquisition (supporting); investigation (supporting); resources (equal). **Shu‐Dong Wei:** Conceptualization (equal); investigation (equal); project administration (equal); resources (supporting); supervision (supporting); writing – review and editing (supporting). **Hui Chen:** Conceptualization (equal); data curation (equal); funding acquisition (lead); investigation (equal); methodology (equal); project administration (equal); resources (equal); supervision (equal); visualization (equal); writing – review and editing (lead).

## FUNDING INFORMATION

This research was financially supported by the Science and Technology Research Project from the Hubei Provincial Department of Education (D20221301), the Engineering Research Center of Ecology and Agricultural Use of Wetland, Ministry of Education (Yangtze University) (KFT202101), the Opening Project of Henan Province Key Laboratory of Water Pollution Control and Rehabilitation Technology (CJSP2022003), and the Innovation and Entrepreneurship Training Plan for Undergraduate in Hubei Province (S202010489074, Yz2022227).

## CONFLICT OF INTEREST

The author declares no conflict of interest.

## Data Availability

The data that support the findings of this study are openly available in Dryad at https://doi.org/10.5061/dryad.sqv9s4n7c.

## References

[ece39702-bib-0001] Anderson, R. C. , Nelson, D. , Anderson, M. R. , & Rickey, M. A. (2005). White‐tailed deer (*Odocoileus virginianus* Zimmermann) browsing effects on tallgrass prairie forbs: Diversity and species abundances. Natural Areas Journal, 25(1), 19–25. 10.3159/TORREY-D-15-00024.1

[ece39702-bib-0002] Ayliffe, L. K. , Cerling, T. E. , Robinson, T. , West, A. G. , Sponheimer, M. , Passey, B. H. , Hammer, J. , Roeder, B. , Dearing, M. D. , & Ehleringer, J. R. (2004). Turnover of carbon isotopes in tail hair and breath CO_2_ of horses fed an isotopically varied diet. Oecologia, 139(1), 11–22. 10.1007/s00442-003-1479-x 14730442

[ece39702-bib-0003] Azorit, C. , Tellado, S. , Oya, A. , & Moro, J. (2012). Seasonal and specific diet variations in sympatric red and fallow deer of southern Spain: A preliminary approach to feeding behaviour. Animal Production Science, 52(8), 720–727. 10.1071/an12016

[ece39702-bib-0004] Bahar, B. , Harrison, S. M. , Moloney, A. P. , Monahan, F. J. , & Schmidt, O. (2014). Isotopic turnover of carbon and nitrogen in bovine blood fractions and inner organs. Rapid Communications in Mass Spectrometry, 28(9), 1011–1018. 10.1002/rcm.6872 24677522

[ece39702-bib-0005] Bao, F. , & Shi, F. C. (2007). Comparative study on physiological characteristics between an invasive plant *Spartina alterniflora* and indigenous plant phragmites communis. Bulletin of Botanical Research, 27(4), 421–427. 10.7525/j.issn.1673-5102.2007.04.010

[ece39702-bib-0006] Beest, F. M. V. , Mysterud, A. , Loe, L. E. , & Milner, J. M. (2010). Forage quantity, quality and depletion as scale‐dependent mechanisms driving habitat selection of a large browsing herbivore. Journal of Animal Ecology, 79(4), 910–922. 10.1111/j.1365-2656.2010.01701.x 20443990

[ece39702-bib-0007] Beever, D. , Terry, R. , Cammell, S. , & Wallace, A. (1978). The digestion of spring and autumn harvested perennial ryegrass by sheep. The Journal of Agricultural Science, 90(3), 463–470. 10.1017/S0021859600055970

[ece39702-bib-0008] Bergvall, U. A. , & Balogh, A. C. V. (2009). Consummatory simultaneous positive and negative contrast in fallow deer: Implications for selectivity. Mammalian Biology, 74, 238–241. 10.1016/j.mambio.2008.05.004

[ece39702-bib-0009] Bergvall, U. A. , & Leimar, O. (2005). Plant secondary compounds and the frequency of food types affect food choice by mammalian herbivores. Ecology, 86, 2450–2460. 10.1890/04-0978

[ece39702-bib-0010] Berteaux, D. , Crête, M. , Huot, J. , Maltais, J. , & Ouellet, J. P. (1998). Food choice by white‐tailed deer in relation to protein and energy content of the diet: A field experiment. Oecologia, 115, 84–92. 10.1007/s004420050494 28308471

[ece39702-bib-0011] Boggs, C. L. (2009). Understanding insect life histories and senescence through a resource allocation lens. Functional Ecology, 23(1), 27–37. 10.1111/j.1365-2435.2009.01527.x

[ece39702-bib-0012] Bond, A. L. , & Diamond, A. W. (2011). Recent Bayesian stable‐isotope mixing models are highly sensitive to variation in discrimination factors. Ecological Applications, 21(4), 1017–1023. 10.1890/09-2409.1 21774408

[ece39702-bib-0117] Bouma, T. J. , van Belzen, J. , Balke, T. , Zhu, Z. , Airoldi, L. , Blight, A. J. , Davies, A. J. , Galvan, C. , Hawkins, S. J. , Hoggart, S. P. G. , Lara, J. L. , Losada, I. J. , Maza, M. , Ondiviela, B. , Skov, M. W. , Strain, E. M. , Thompson, R. C. , Yang, S. , Zanuttigh, B. , … Herman, P. M. J. (2014). Identifying knowledge gaps hampering application of intertidal habitats in coastal protection: Opportunities & steps to take. Coastal Engineering, 87, 147–157. 10.1016/j.coastaleng.2013.11.014

[ece39702-bib-0013] Campos‐Arceiz, A. , & Takatsuki, S. (2005). Food habits of sika deer in the Shiranuka Hills, eastern Hokkaido: A northern example from the north–south variations in food habits in sika deer. Ecological Research, 20(2), 129–133. 10.1007/s11284-004-0019-4

[ece39702-bib-0014] Cao, K. Q. (1985). Discussion on the causes of wild *Elaphurus davidianus* extinction. Zoological Research, 6(1), 111–115.

[ece39702-bib-0015] Caut, S. , Angulo, E. , & Courchamp, F. (2009). Variation in discrimination factors (Δ^15^N and Δ^13^C): The effect of diet isotopic values and applications for diet reconstruction. Journal of Applied Ecology, 46(2), 443–453. 10.1111/j.1365-2664.2009.01620.x

[ece39702-bib-0016] Cerling, T. E. , Wittemyer, G. , Rasmussen, H. B. , Vollrath, F. , Cerling, C. E. , Robinson, T. J. , & Douglas‐Hamilton, I. (2006). Stable isotopes in elephant hair document migration patterns and diet changes. Proceedings of the National Academy of Sciences of the United States of America, 103(2), 371–373. 10.1073/pnas.0509606102 16407164 PMC1326175

[ece39702-bib-0017] Champagne, E. , Royo, A. A. , Tremblay, J. P. , & Raymond, P. (2020). Phytochemicals involved in plant resistance to leporids and Cervids: A systematic review. Journal of Chemical Ecology, 46(1), 84–98. 10.1007/s10886-019-01130-z 31858366

[ece39702-bib-0018] Chapman, G. A. , Bork, E. W. , Donkor, N. T. , & Hudson, R. J. (2010). Effects of supplemental dietary tannins on the performance of white‐tailed deer (*Odocoileus virginianus*). Journal of Animal Physiology and Animal Nutrition, 94, 65–73. 10.1111/j.1439-0396.2008.00883.x 19364384

[ece39702-bib-0019] Cui, D. Y. , Liu, Z. S. , Wang, X. M. , Zha, H. , Hu, T. H. , & Li, Z. G. (2007). Winter food‐habits of Red Deer (*Cervus elaphus alxaicus*) in Helan mountains, China. Zoologica Research, 28(4), 383–388. 10.3321/j.issn:0254-5853.2007.04.007

[ece39702-bib-0020] Davidson, N. C. , Fluet‐Chouinard, E. , & Finlayson, C. M. (2018). Global extent and distribution of wetlands: Trends and issues. Marine and Freshwater Research, 69(4), 620–627. 10.1071/MF17019

[ece39702-bib-0021] De Smet, S. , Balcaen, A. , Claeys, E. , Boeckx, P. , & Van Cleemput, O. (2004). Stable carbon isotope analysis of different tissues of beef animals in relation to their diet. Rapid Communications in Mass Spectrometry, 18(11), 1227–1232. 10.1002/rcm.1471 15164353

[ece39702-bib-0022] Demment, M. W. , & Soest, P. J. V. (1985). A nutritional explanation for body‐size patterns of ruminant and nonruminant herbivores. The American Naturalist, 125(5), 641–672. 10.1086/284369

[ece39702-bib-0023] Ding, Y. H. (2004). Studies on Milu (Elaphurus davidianus) in China. Jilin Science and Technology Press.

[ece39702-bib-0024] Ding, Y. H. (2009). Preference of feeding on *Spartina alterniflora* loisel by Mi‐deer in Dafeng National Nature Reserve. Chinese Journal of Wildlife, 30(3), 118–120. 10.19711/j.cnki.issn2310-1490.2009.03.002

[ece39702-bib-0025] Ding, Y. H. (2017). David's deer. Nanjing Normal University Press.

[ece39702-bib-0026] Ding, Y. H. , Liu, G. X. , Sun, D. M. , & Zhou, Y. S. (1989). Preliminary study on forage plants of Milu. Animal Husbandry & Veterinary Medicine, 40(3), 116–117.

[ece39702-bib-0027] Dumont, A. , Ouellet, J.‐P. , Crête, M. , & Huot, J. (2005). Winter foraging strategy of white‐tailed deer at the northern limit of its range. Écoscience, 12(4), 476–484. 10.2980/i1195-6860-12-4-476.1

[ece39702-bib-0028] Ehrich, D. , Tarroux, A. , Stien, J. , Lecomte, N. , Killengreen, S. , Berteaux, D. , & Yoccoz, N. G. (2011). Stable isotope analysis: Modelling lipid normalization for muscle and eggs from arctic mammals and birds. Methods in Ecology and Evolution, 2(1), 66–76. 10.1111/j.2041-210X.2010.00047.x

[ece39702-bib-0029] Endo, Y. , Takada, H. , & Takatsuki, S. (2017). Comparison of the food habits of the sika deer (*Cervus nippon*), the Japanese Serow (*Capricornis crispus*), and the wild boar (*Sus scrofa*), sympatric herbivorous mammals from Mt. Asama, Central Japan. Mammal Study, 42(3), 131–140. 10.3106/041.042.0303

[ece39702-bib-0030] Estell, R. E. (2010). Coping with shrub secondary metabolites by ruminants. Small Ruminant Research, 94(1–3), 1–9. 10.1016/j.smallrumres.2010.09.012

[ece39702-bib-0031] Fan, J. , Wang, X. D. , Wu, W. , Chen, W. P. , Ma, Q. , & Ma, Z. J. (2021). Function of restored wetlands for waterbird conservation in the Yellow Sea coast. Science of the Total Environment, 756, 144061. 10.1016/j.scitotenv.2020.144061 33280877

[ece39702-bib-0032] Felton, A. M. , Felton, A. , Raubenheimer, D. , Simpson, S. J. , Krizsan, S. J. , Hedwall, P.‐O. , & Stolter, C. (2016). The nutritional balancing act of a large herbivore: An experiment with captive moose (*Alces alces* L.). PLoS One, 11(3), e0150870. 10.1371/journal.pone.0150870 26986618 PMC4795764

[ece39702-bib-0033] Folch, J. , Lees, M. , & Stanley, G. H. S. (1957). A simple method for the isolation and purification of total lipides from animal tissues. Journal of Biological Chemistry, 226(1), 497–509. 10.1016/S0021-9258(18)64849-5 13428781

[ece39702-bib-0035] Gebert, C. , & Verheyden‐Tixier, H. (2001). Variations of diet composition of Red Deer (*Cervus elaphus* L.) in Europe. Mammal Review, 31(3–4), 189–201. 10.1111/j.1365-2907.2001.00090.x

[ece39702-bib-0036] Guo, B. L. , Wei, Y. M. , & Pan, J. R. (2008). Study on the changing law of stable carbon isotope composition in cattle tail hair. Scientia Agricultura Sinica, 7, 2105–2111. 10.3864/j.issn.0578-1752.2008.07.031

[ece39702-bib-0037] Hayes, M. (2020). Measuring protein content in food: An overview of methods. Foods, 9(10), 1340. 10.3390/foods9101340 32977393 PMC7597951

[ece39702-bib-0038] Hobbs, N. T. , Baker, D. L. , & Gill, R. B. (1983). Comparative nutritional ecology of montane ungulates during winter. The Journal of Wildlife Management, 47(1), 1–16. 10.2307/3808046

[ece39702-bib-0039] Hobson, K. A. , Mclellan, B. , & Woods, J. G. (2000). Using stable carbon (δ^13^C) and nitrogen (δ^15^N) isotopes to infer trophic relationships among black and grizzly bears in the upper Columbia River basin, British Columbia. Canadian Journal of Zoology, 78, 1332–1339. 10.1007/s00442-022-05257-x

[ece39702-bib-0040] Hua, R. , Cui, D. Y. , Liu, J. , Li, S. H. , Zhao, Y. Q. , & Zhang, Y. N. (2020). Winter diets of Père David's deer in Yancheng wetland, Jiangsu Province, China. Chinese Journal of Zoology, 55(1), 1–8. 10.13859/j.cjz.202001001

[ece39702-bib-0041] Hummel, S. L. , Campa, H., III , & Winterstein, S. R. (2018). Understanding how a keystone herbivore, white‐tailed deer impacts wetland vegetation types in southern Michigan. American Midland Naturalist, 179(1), 51–67. 10.1674/0003-0031-179.1.51

[ece39702-bib-0042] Iijima, H. , Nagaike, T. , & Honda, T. (2013). Estimation of deer population dynamics using a bayesian state‐space model with multiple abundance indices. Journal of Wildlife Management, 77(5), 1038–1047. 10.1002/jwmg.556

[ece39702-bib-0043] Illius, A. W. , & Gordon, I. J. (1990). Constraints on diet selection and foraging behaviour in mammalian herbivores. In R. N. Hughes (Ed.), Behavioural mechanisms of food selection. NATO ASI series (Vol. 20). Springer. 10.1007/978-3-642-75118-919

[ece39702-bib-0044] Jackson, A. L. , Inger, R. , Bearhop, S. , & Parnell, A. (2008). Erroneous behaviour of MixSIR, a recently published Bayesian isotope mixing model: A discussion of Moore & Semmens. Ecology Letters, 12(3), E1–E5. 10.1111/j.1461-0248.2008.01233.x 18691222

[ece39702-bib-0045] Janne, A. , Marja, L. , Lars, B. S. , Jorge, G. G. , Maija, T. , Jani, H. , & Kevin, M. (2021). Macroecology of macrophytes in the freshwater realm: Patterns, mechanisms and implications. Aquatic Botany, 168, 103325. 10.1016/j.aquabot.2020.103325

[ece39702-bib-0046] Jarman, P. J. (1974). The social organisation of antelope in relation to their ecology. Behaviour, 48, 215–267.

[ece39702-bib-0047] Ji, Y. F. , Wu, B. L. , Ding, Y. H. , & Qin, P. (2011). Nutritional components of *Phragmites australis* and *Spartina alterniflora* in Dafeng free‐range David's deer habitat of Jiangsu Province, East China: A comparative analysis. Chinese Journal of Ecology, 30(10), 2240–2244. 10.13292/j.1000-4890.2011.0298

[ece39702-bib-0048] Johnson, C. J. , Parker, K. L. , & Heard, D. C. (2001). Foraging across a variable landscape: Behavioral decisions made by woodland caribou at multiple spatial scales. Oecologia, 127(4), 590–602. 10.1007/s004420000573 28547497

[ece39702-bib-0049] Krojerová‐Prokešová, J. , Barančeková, M. , Šustr, P. , & Heurich, M. (2010). Feeding patterns of red deer *Cervus elaphus* along an altitudinal gradient in the bohemian Forest: Effect of habitat and season. Wildlife Biology, 16(2), 173–184. 10.2981/09-004

[ece39702-bib-0050] Li, C. , Yang, D. D. , Zhang, Y. M. , Song, Y. C. , & Li, P. F. (2015). Autumn nocturnal bed‐site selection by free‐ranging and re‐wilding populations of Milu (*Elaphurus davidianus*) in Shishou County of Hubei Province, China. Chinese Journal of Ecology, 34(10), 2855–2860. 10.13292/j.1000-4890.2015.0270

[ece39702-bib-0051] Li, P. F. , Wen, H. J. , Sha, P. , Zhang, Y. M. , & Yang, T. (2012). Habitat degradation and its conservation strategies in Shishou Milu National Nature Reserve. Journal of Green Science and Technology, 3(6), 249–251. 10.3969/j.issn.1674-9944.2012.06.115

[ece39702-bib-0052] Li, Y. P. , Zhang, S. M. , Liu, Y. J. , & Bai, J. D. (2017). Nutrient composition analysis of elk favorite plants in different habitats. Heilongjiang Animal Science and Veterinary Medicine, 60(17), 252–255. 10.13881/j.cnki.hljxmsy.2017.1569

[ece39702-bib-0053] Li, Z. , Cao, Y. , Fu, W. L. , Wei, W. , Li, P. F. , Wen, H. J. , & Li, W. (2016). Relationship between soil seed bank and above‐ground vegetation in the degraded wetland region of Shishou swan Island elk nature reserve, Hubei province. Journal of Hydroecology, 37(3), 34–41. 10.15928/j.1674-3075.2016.03.005

[ece39702-bib-0054] Maloiy, G. M. O. , Kay, R. N. B. , Goodall, E. D. , & Topps, J. H. (1970). Digestion and nitrogen metabolism in sheep and red deer given large or small amounts of water and protein. British Journal of Nutrition, 24(3), 843–855. 10.1079/bjn19700087 5470784

[ece39702-bib-0055] McCue, M. D. , Javal, M. , Clusella‐Trullas, S. , Le Roux, J. J. , Jackson, M. C. , Ellis, A. G. , Richardson, D. M. , Valentine, A. J. , & Terblanche, J. S. (2020). Using stable isotope analysis to answer fundamental questions in invasion ecology: Progress and prospects. Methods in Ecology and Evolution, 11(2), 196–214. 10.1111/2041-210x.13327

[ece39702-bib-0056] Meng, Y. P. , Li, K. , Zhang, L. Y. , Yang, Y. , & Chen, Y. (2010). Measurement and analysis on feed intake of *Elaphurus davidianus* in Beijing Milupark. Special Wild Economic Animal and Plant Research, 32(4), 39–42. 10.16720/j.cnki.tcyj.2010.04.013

[ece39702-bib-0057] Miranda, M. , Sicilia, M. , Bartolome, J. , Molina‐Alcaide, E. , Galvez‐Bravo, L. , & Cassinello, J. (2012). Contrasting feeding patterns of native red deer and two exotic ungulates in a Mediterranean ecosystem. Wildlife Research, 39(2), 171–182. 10.1071/wr11146

[ece39702-bib-0058] Moore, J. W. , & Semmens, B. X. (2008). Incorporating uncertainty and prior information into stable isotope mixing models. Ecology Letters, 11(5), 470–480. 10.1111/j.1461-0248.2008.01163.x 18294213

[ece39702-bib-0059] Motta, L. , Noelia Barrios‐Garcia, M. , Ballari, S. A. , & Rodriguez‐Cabal, M. A. (2020). Cross‐ecosystem impacts of non‐native ungulates on wetland communities. Biological Invasions, 22(11), 3283–3291. 10.1007/s10530-020-02323-4

[ece39702-bib-0060] Mueller‐Harvey, I. (2006). Unravelling the conundrum of tannins in animal nutrition and health. Journal of the Science of Food and Agriculture, 86(13), 2010–2037. 10.1002/jsfa.2577

[ece39702-bib-0061] Nakamura, M. (1968). Determination of fructose in the presence of a large excess of glucose. Agricultural and Biological Chemistry, 32(6), 689–706. 10.1080/00021369.1968.10859121

[ece39702-bib-0062] O'Hare, M. T. , Aguiar, F. C. , Asaeda, T. , Bakker, E. S. , Chambers, P. A. , Clayton, J. S. , Elger, A. , Ferreira, T. M. , Gross, E. M. , Gunn, I. D. M. , Gurnell, A. M. , Hellsten, S. , Hofstra, D. E. , Li, W. , Mohr, S. , Puijalon, S. , Szoszkiewicz, K. , Willby, N. J. , & Wood, K. A. (2018). Plants in aquatic ecosystems: Current trends and future directions. Hydrobiologia, 812, 1–11. 10.1007/s10750-017-3190-7

[ece39702-bib-0063] O'Regan, H. J. , Chenery, C. , Lamb, A. L. , Stevens, R. E. , Rook, L. , & Elton, S. (2008). Modern macaque dietary heterogeneity assessed using stable isotope analysis of hair and bone. Journal of Human Evolution, 55(4), 617–626. 10.1016/j.jhevol.2008.05.001 18599109

[ece39702-bib-0064] Parnell, A. C. , Inger, R. , Bearhop, S. , & Jackson, A. L. (2010). Source partitioning using stable isotopes: Coping with too much variation. PLoS One, 5(3), e9672. 10.1371/journal.pone.0009672 20300637 PMC2837382

[ece39702-bib-0065] Parnell, A. C. , Phillips, D. L. , Bearhop, S. , Semmens, B. X. , Ward, E. J. , Moore, J. W. , Jackson, A. L. , Grey, J. , Kelly, D. J. , & Inger, R. (2013). Bayesian stable isotope mixing models. Environmetrics, 24(6), 387–399. 10.1002/env.2221

[ece39702-bib-0066] Perrin, B. , & Campbell, M. R. (1980). Key to the mammals of the Andries Vosloo kudu reserve (eastern cape), based on their hair morphology, for use in predator scat analysis. South African Journal of Wildlife Research ‐ 24‐month delayed open access, 10, 1–14. https://hdl.handle.net/10520/AJA03794369_2884

[ece39702-bib-0067] Phillips, D. L. , & Gregg, J. W. (2003). Source partitioning using stable isotopes: Coping with too many sources. Oecologia, 136(2), 261–269. 10.1007/s00442-003-1218-3 12759813

[ece39702-bib-0068] Phillips, D. L. , Inger, R. , Bearhop, S. , Jackson, A. L. , Moore, J. W. , Parnell, A. C. , Semmens, B. X. , & Ward, E. J. (2014). Best practices for use of stable isotope mixing models in food‐web studies. Canadian Journal of Zoology, 92(10), 823–835. 10.1139/cjz-2014-0127

[ece39702-bib-0069] Qian, Y. H. , Wang, L. , & Chen, H. Q. (2008). Research on ecological habitat problem in and the countermeasures for Dafeng moose's stocking. Journal of Library and Information Science, 25(32), 98–99. 10.3969/j.issn.1001-9960.2008.14.284

[ece39702-bib-0070] Qiu, Q. H. (2016). Research progress on effects of condensed tannins on rumen fermentation. China Dairy Cattle, 34(5), 10–13. 10.19305/j.cnki.11-3009/s.2016.05.003

[ece39702-bib-0071] Rioux, È. , Pelletier, F. , & St‐Laurent, M. H. (2020). From diet to hair and blood: Empirical estimation of discrimination factors for C and N stable isotopes in five terrestrial mammals. Journal of Mammalogy, 101(5), 1332–1344. 10.1093/jmammal/gyaa108

[ece39702-bib-0072] Rioux, È. , Pelletier, F. , & St‐Laurent, M.‐H. (2019). Influence of lipids on stable isotope ratios in mammal hair: Highlighting the importance of validation. Ecosphere, 10(5), e02723. 10.1002/ecs2.2723

[ece39702-bib-0073] Rogers, M. C. , Hilderbrand, G. V. , Gustine, D. D. , Joly, K. , Leacock, W. B. , Mangipane, B. A. , & Welker, J. M. (2020). Splitting hairs: Dietary niche breadth modelling using stable isotope analysis of a sequentially grown tissue. Isotopes in Environmental and Health Studies, 56(4), 358–369. 10.1080/10256016.2020.1787404 32631088

[ece39702-bib-0074] Roth, J. D. , & Hobson, K. A. (2000). Stable carbon and nitrogen isotopic fractionation between diet and tissue of captive seals: Implications for dietary reconstructions involving marine mammals. Canadian Journal of Zoology, 78(5), 848–852. 10.1139/cjz-78-5-848

[ece39702-bib-0075] Saito, L. , Johnson, B. M. , Bartholow, J. , & Hanna, R. B. (2001). Assessing ecosystem effects of reservoir operations using food web–energy transfer and water quality models. Ecosystems, 4(2), 105–125. 10.1007/s100210000062

[ece39702-bib-0076] Schingoethe, D. J. , Skyberg, E. W. , & Rook, J. A. (1980). Chemical composition of sunflower silage as influenced by additions of urea, dried whey and sodium hydroxide. Journal of Animal Science, 50, 625–629.

[ece39702-bib-0077] Schwertl, M. , Auerswald, K. , Schaufele, R. , & Schnyder, H. (2005). Carbon and nitrogen stable isotope composition of cattle hair: Ecological fingerprints of production systems? Agriculture Ecosystems & Environment, 109(1–2), 153–165. 10.1016/j.agee.2005.01.015

[ece39702-bib-0078] Schwertl, M. , Auerswald, K. , & Schnyder, H. (2003). Reconstruction of the isotopic history of animal diets by hair segmental analysis. Rapid Communications in Mass Spectrometry, 17(12), 1312–1318. 10.1002/rcm.1042 12811754

[ece39702-bib-0079] Seto, T. , Matsuda, N. , Okahisa, Y. , & Kaji, K. (2015). Effects of population density and snow depth on the winter diet composition of sika deer. Journal of Wildlife Management, 79(2), 243–253. 10.1002/jwmg.830

[ece39702-bib-0081] Smith, J. M. (1978). Optimization theory in evolution. Annual Review of Ecology, Evolution, and Systematics, 9(1), 31–56. 10.1146/annurev.es.09.110178.000335

[ece39702-bib-0082] Soest, P. J. V. (2018). Nutritional ecology of the ruminant. Cornell University Press.

[ece39702-bib-0083] Sorensen, J. S. , McLister, J. D. , & Dearing, M. D. (2005). Plant secondary metabolites compromise the energy budgets of specialist and generalist mammalian herbivores. Ecology, 86(1), 125–139. 10.1890/03-0627

[ece39702-bib-0084] Sponheimer, M. , Robinson, T. F. , Cerling, T. E. , Tegland, L. , Roeder, B. L. , Ayliffe, L. , Dearing, M. D. , & Ehleringer, J. R. (2006). Turnover of stable carbon isotopes in the muscle, liver, and breath CO_2_ of alpacas (*Lama pacos*). Rapid Communications in Mass Spectrometry, 20(9), 1395–1399. 10.1002/rcm.2454 16572383

[ece39702-bib-0085] Steele, K. W. , & Daniel, R. M. (1978). Fractionation of nitrogen isotopes by animals: A further complication to the use of variations in the natural abundance of ^15^ N for tracer studies. The Journal of Agricultural Science, 90(1), 7–9. 10.1017/S002185960004853X

[ece39702-bib-0086] Stephens, R. B. , Ouimette, A. P. , Hobbie, E. A. , & Rowe, R. J. (2022). Reevaluating trophic discrimination factors (δ^13^C and δ^15^N) for diet reconstruction. Ecological Monographs, 92, e1525. 10.1002/ecm.1525

[ece39702-bib-0087] Taillon, J. , Sauvë, D. G. , & Côté, S. D. (2006). The effects of decreasing winter diet quality on foraging behavior and life‐history traits of white‐tailed deer fawns. The Journal of Wildlife Management, 70(5), 1445–1454. 10.2193/0022-541X(2006)70[1445:TEODWD]2.0.CO;2

[ece39702-bib-0088] Takatsuki, S. (2009). Geographical variations in food habits of sika deer: The northern grazer vs. the southern browser. In D. R. McCullough , S. Takatsuki , & K. Kaji (Eds.), Sika deer: Biology and management of native and introduced populations (pp. 231–237). Springer Japan.

[ece39702-bib-0089] Tieszen, L. L. , Boutton, T. W. , Tesdahl, K. G. , & Slade, N. A. (1983). Fractionation and turnover of stable carbon isotopes in animal tissues: Implications for δ^13^C analysis of diet. Oecologia, 57(1), 32–37. 10.1007/BF00379558 28310153

[ece39702-bib-0090] Verheyden‐Tixier, H. , & Duncan, P. (2000). Selection for small amounts of hydrolysable tannins by a concentrate‐selecting mammalian herbivore. Journal of Chemical Ecology, 26, 351–358. 10.1023/A:1005401203954

[ece39702-bib-0091] Verheyden‐Tixier, H. , Renaud, P.‐C. , Morellet, N. , Jamot, J. , Besle, J.‐M. , & Dumont, B. (2008). Selection for nutrients by red deer hinds feeding on a mixed forest edge. Oecologia, 156(3), 715–726. 10.1007/s00442-008-1020-3 18357470

[ece39702-bib-0092] Wang, L. B. , Jiang, H. , An, Y. T. , Yang, Y. Z. , & Yuan, B. D. (2020). Current status and conservation measures for Père David's deer in China. Chinese Journal of Wildlife, 41(3), 806–813. 10.19711/j.cnki.issn2310-1490.2020.03.034

[ece39702-bib-0093] Wang, Q. , Wang, C. H. , Zhao, B. , Ma, Z. J. , Luo, Y. Q. , Chen, J. K. , & Li, B. (2006). Effects of growing conditions on the growth of and interactions between salt marsh plants: Implications for invasibility of habitats. Biological Invasions, 8(7), 1547–1560. 10.1007/s10530-005-5846-x

[ece39702-bib-0094] Wang, Y. , & Wang, W. (2011). Diet of Père David's deer (*Elaphurus davidianus*) at Milu Park in Beijing, China. Chinese Journal of Wildlife, 32(2), 65–68. 10.19711/j.cnki.issn2310-1490.2011.02.002

[ece39702-bib-0095] Wei, S. D. , Chen, H. , Yan, T. , Lin, Y. M. , & Zhou, H. C. (2014). Identification of antioxidant components and fatty acid profiles of the leaves and fruits from *Averrhoa carambola* . LWT‐Food Science and Technology, 55(1), 278–285. 10.1016/j.lwt.2013.08.013

[ece39702-bib-0096] Windschitl, P. M. , & Stern, M. D. (1988). Influence of methionine derivatives on effluent flow of methionine from continuous culture of ruminal bacteria. Journal of Animal Science, 66(11), 2937–2947. 10.2527/jas1988.66112937x 3225247

[ece39702-bib-0097] Wolka, K. , Mulder, J. , & Biazin, B. (2018). Effects of soil and water conservation techniques on crop yield, runoff and soil loss in sub‐Saharan Africa: A review. Agricultural Water Management, 207, 67–79. 10.1016/j.agwat.2018.05.016

[ece39702-bib-0098] Workman, C. , & Schmitt, D. (2012). Positional behavior of delacour's langurs (*Trachypithecus delacouri*) in northern Vietnam. International Journal of Primatology, 33(1), 19–37. 10.1007/s10764-011-9547-2

[ece39702-bib-0099] Xu, A. H. , & Yu, X. P. (2019). Conservation status and sustainable development strategy of Milu (*Elaphurus davidianus*) in Dafeng. Journal of Anhui Agricultural Sciences, 47(5), 107–109. 10.3969/j.issn.0517-6611.2019.05.029

[ece39702-bib-0100] Xue, D. Y. , Zhang, Y. Y. , Cheng, Z. B. , Zhong, Z. Y. , Cao, M. , Fu, M. D. , Bai, J. , & Yuan, X. J. (2022). Père David's deer (*Elaphurus davidianus*) in China: Population dynamics and challenges. Journal of Resources and Ecology, 13(1), 41–50. 10.5814/j.issn.1674-764x.2022.01.005

[ece39702-bib-0101] Yousef Elahi, M. , & Rouzbehan, Y. (2008). Characteriztion of *Quercus persica*, *Quercus infectoria* and *Quercus libani* as ruminant feeds. Animal Feed Science and Technology, 140(1), 78–89. 10.1016/j.anifeedsci.2007.02.009

[ece39702-bib-0102] Zhang, H. S. , Ai, J. S. , Wen, H. J. , Zhang, Y. M. , Li, P. F. , Yang, T. , & Zhu, J. Q. (2018). The environmental status and protection of the habitat of Shishou elk. Advances in Meteorological Science and Technology, 8(5), 109–112. 10.3969/j.issn.2095-1973.2018.05.030

[ece39702-bib-0103] Zhang, H. S. , Li, P. F. , Wen, H. J. , Tian, G. M. , Chen, H. , Zhang, L. , & Zhu, J. Q. (2019). Population status and research progress of pere David's deer (*Elaphurus davidianus*) in China. Pakistan Journal of Zoology, 51(6), 2359–2367. 10.17582/journal.pjz/2019.51.6.rev1

[ece39702-bib-0104] Zhang, H. S. , Tian, G. M. , Wang, X. Y. , Li, P. F. , & Zhu, J. Q. (2020). Habitat selection of milu (*Elaphurus davidianus*) in Shishou in the dry season of Yangtze River. Chinese Journal of Wildlife, 41(1), 22–28. 10.19711/j.cnki.issn2310-1490.2020.01.004

[ece39702-bib-0105] Zhang, J. M. , Fu, L. F. , Hong, X. , Wang, L. , Wang, Z. , & Zhou, S. B. (2019). Herbaceous flora and species diversity in Shishou Milu National Nature Reserve. Chinese Journal of Ecology, 38(2), 513–520. 10.13292/j.1000-4890.201902.025

[ece39702-bib-0106] Zhang, L. H. , Zen, C. S. , & Quan, C. (2008). Study on biomass dynamics of *Phragmites australis* and *Spartina alterniflora* in the wetlands of Minjiang River estuary. Journal of Subtropical Resources and Environment, 23(2), 25–33. 10.19687/j.cnki.1673-7105.2008.02.004

[ece39702-bib-0107] Zhang, X. J. (2015). Study on feeding habits of wild free‐range elk in Dafeng and analysis of plant nutrition of its staple food (Master). Nanjing Normal University.

[ece39702-bib-0108] Zhang, Y. , Bai, J. , Zhu, A. , Chen, R. , Xue, D. , Zhong, Z. , & Cheng, Z. (2021). Reversing extinction in China's pere David's deer. Science, 371(6530), 685. 10.1126/science.abg6237 33574204

[ece39702-bib-0109] Zhang, Y. , Wang, M. , Xiao, Z. H. , Tang, Y. , Fang, F. , Li, P. F. , & Liu, S. X. (2013). Land use dynamic change analysis based on “3 S” technology in Shishou David's deer National Nature Reserve. Forest Inventory and Planning, 38(3), 16–20.

[ece39702-bib-0110] Zhang, Y. M. , & Yang, T. (2017). Shishou Milu national nature reserve, Atlas of common birds and plants. Jiangsu Phoenix Science and Technology Publishing Press.

[ece39702-bib-0111] Zhao, X. L. , Lin, Y. , Zhang, G. F. , Xie, S. B. , Hua, W. J. , & Ding, Y. H. (2010). Community characteristics of beach wetland vegetations along a habitat gradient in Dafeng Milu Reserve of Jiangsu Province. Chinese Journal of Ecology, 29(2), 244–249. 10.13292/j.1000-4890.2010.0007

[ece39702-bib-0112] Zhong, L. Q. , Zhang, W. Q. , Yang, M. , Wu, S. Y. , Zhi, X. L. , & Zhang, M. H. (2019). Winter diet variation and overlap of sympatric red deer and sika deer in Northeast China. Polish Journal of Ecology, 67(4), 354–366. 10.3161/15052249pje2019.67.4.007

[ece39702-bib-0114] Zhou, C. Y. , Fei, Y. J. , Wu, L. , & Yang, C. D. (2010). Effects of elk grazing on soil physical and chemical properties of grassland on Tiane Island. Acta Prataculturae Sinica, 19(4), 115–121. 10.11686/cyxb20100416

[ece39702-bib-0115] Zou, S. J. , Song, Y. C. , Yang, D. D. , & Li, P. F. (2013). Winter bed‐site microhabitat selection by pere David' s deer (*Elaphurus davidianus*) in Hubei Shishou Milu National Nature Reserve, south‐Central China. Chinese Journal of Ecology, 32(4), 899–904. 10.13292/j.1000-4890.2013.0190

[ece39702-bib-0116] Zou, W. , Lusk, C. , Messer, D. , & Lane, R. (1999). Fat contents of cereal foods: Comparison of classical with recently developed extraction techniques. Journal of AOAC International, 82(1), 141–150. 10028683

